# Giant Tumor in the Inferior Vena Cava Treated With CyberKnife

**DOI:** 10.7759/cureus.13620

**Published:** 2021-02-28

**Authors:** Yuko Harada, Shinichiro Miyazaki, Toshiaki Kunimura

**Affiliations:** 1 Cardiology, Kawasaki Municipal Ida Hospital, Kawasaki, JPN; 2 Radiation Oncology, CyberKnife Center, Shin-Yurigaoka General Hospital, Kawasaki, JPN; 3 Clinical Pathology, Yamato Tokushukai Hospital, Yamato, JPN

**Keywords:** ivc tumor, cyberknife, radiotherapy, clear cell renal cell carcinoma

## Abstract

Renal cell carcinoma (RCC) is a slow-progressing cancer that may cause tumor embolism in the inferior vena cava (IVC) and has a high mortality rate. Treatment for IVC metastasis of RCC is basically surgical resection often requiring cardiopulmonary bypass. RCC has been regarded as a radio-resistant tumor; however, stereotactic radiotherapy (SRT) has proven effective in recent years. We present a case of advanced RCC in which CyberKnife radiotherapy was successful in saving and preserving quality of life.

An 81-year-old male presented with severe edema in both legs. Contrast CT scan displayed giant tumor in IVC and bilateral mediastinal lymphadenopathy. The cancer appeared to originate from the lower pole of the right kidney. The tumor protruded into the right atrium, and surgical resection with pump oxygenator was impossible due to patient’s age. CyberKnife SRT was performed for tumor in the IVC. Biopsy for hilar lymph node revealed clear cell RCC, and the second CyberKnife treatment was performed. The patient is surviving over three years without any symptoms. CyberKnife was successful in preserving patient’s quality of life for advanced stage IV RCC.

## Introduction

Renal cell carcinoma (RCC) is a slow-progressing cancer. The mortality rate of RCC is not considered high; however, RCC could be fatal if it invades large vessels. The first choice of treatment is surgical resection. Radiotherapy is not considered effective. Molecular targeted drugs are used for inoperative cases. Treatment options are very limited for elderly patients with advanced stage RCC. Patients notice symptoms usually at advanced stage. Most patients are elderly and cannot tolerate surgery or chemotherapy. In recent years, radiotherapy has attracted attention as a promising treatment for advanced RCC.

CyberKnife (Accuray Incorporated, Sunnyvale, CA, USA) is a robotic radiosurgery system that provides highly precise stereotactic radiotherapy (SRT). SRT with CyberKnife is minimally invasive and is usually completed within several days. Thus, CyberKnife is the preferred option for palliative treatment of advanced cancer.

In the case presented here, giant tumor of RCC in the inferior vena cava (IVC) was successfully treated with CyberKnife. SRT by CyberKnife demonstrated successful to sustain patient’s life.

## Case presentation

**Figure 1:**
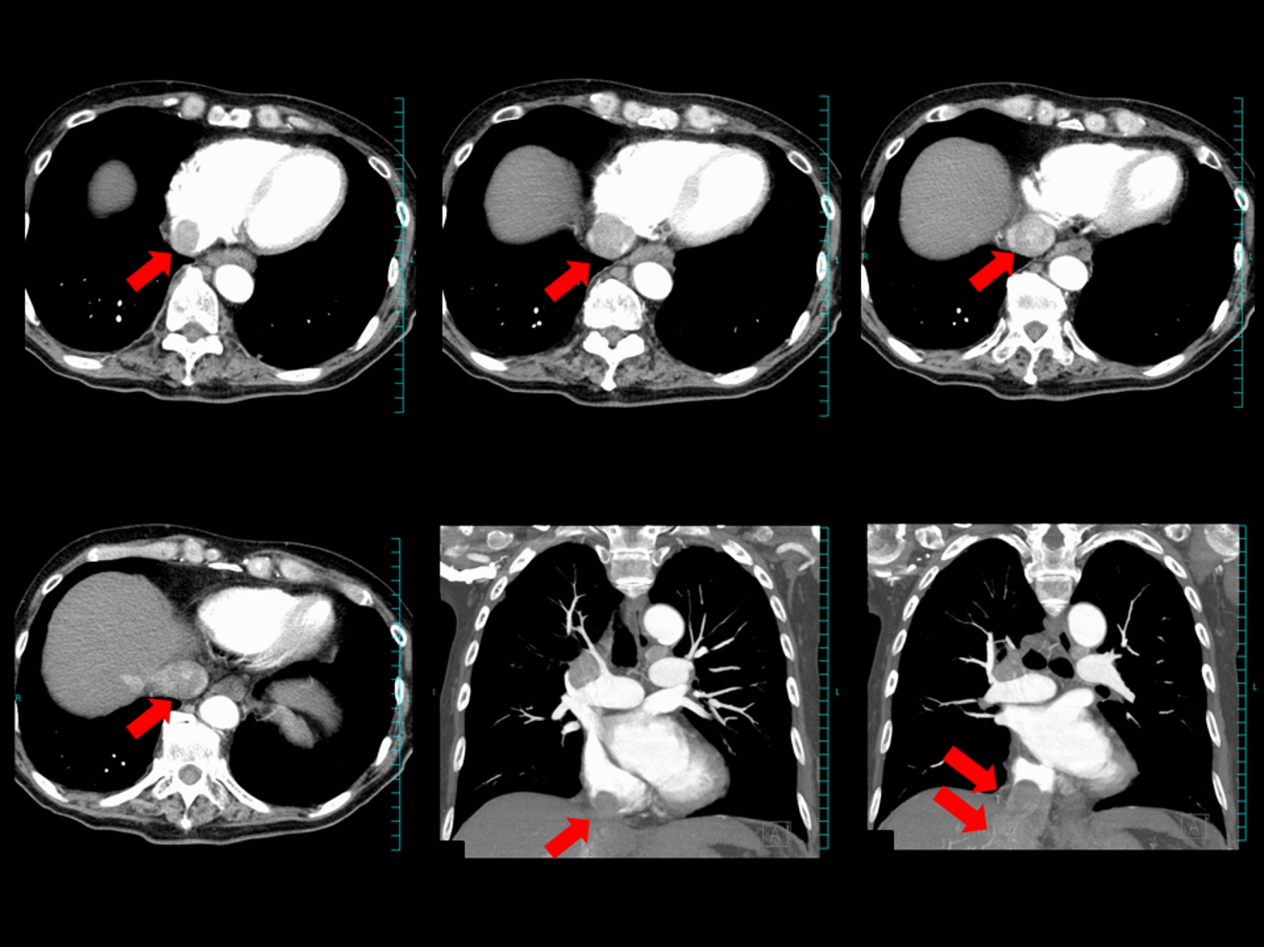
Contrast CT scan at diagnosis. Massive IVC tumor (red arrow) is protruding into the right atrium. IVC, inferior vena cava

**Figure 2:**
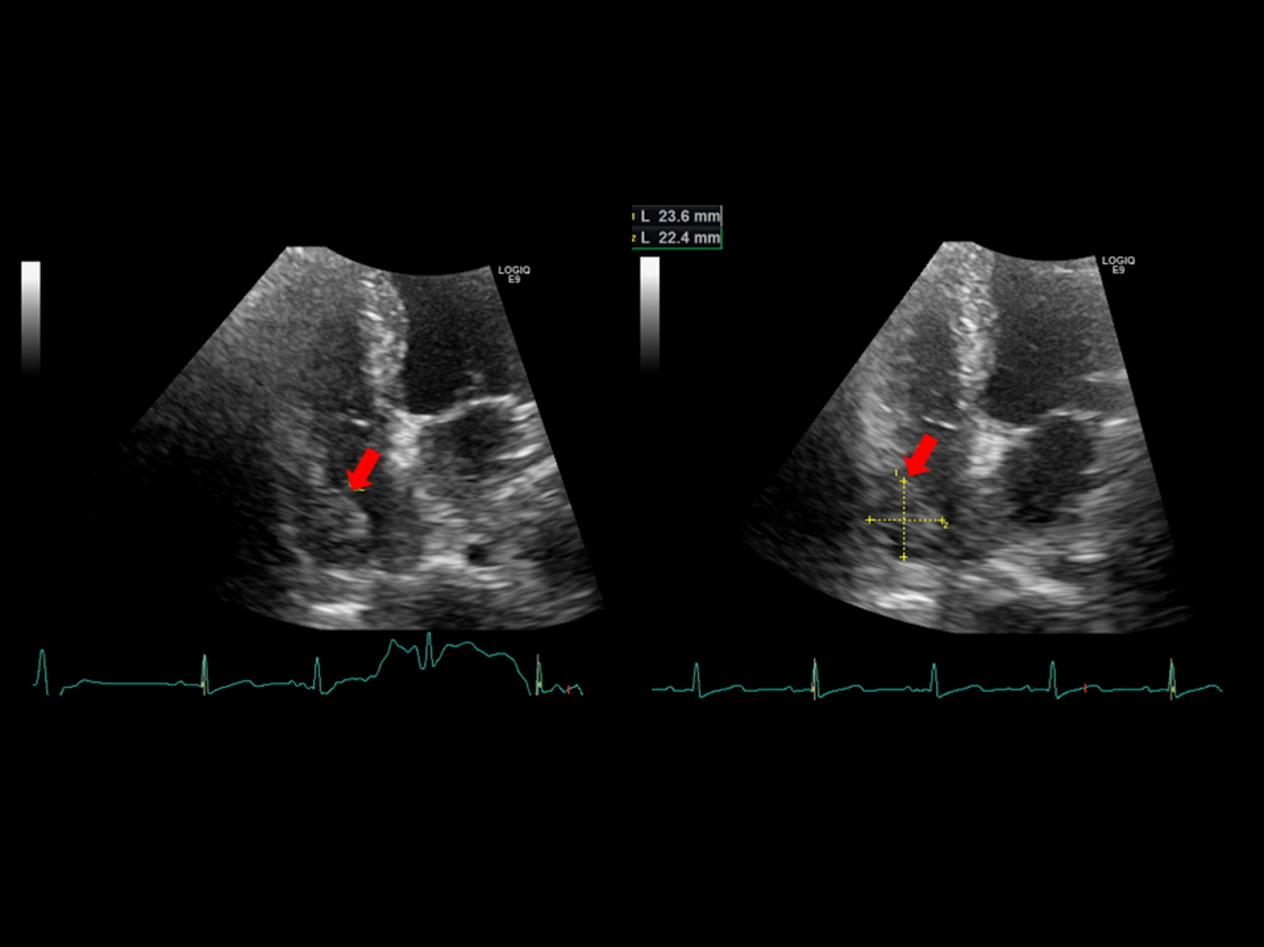
Cardiac ultrasound showing tumor. Four-chamber view from the apex. Red arrow denotes IVC tumor protruding into the right atrium (23.6 x 22.4 mm in size). IVC, inferior vena cava

**Figure 3:**
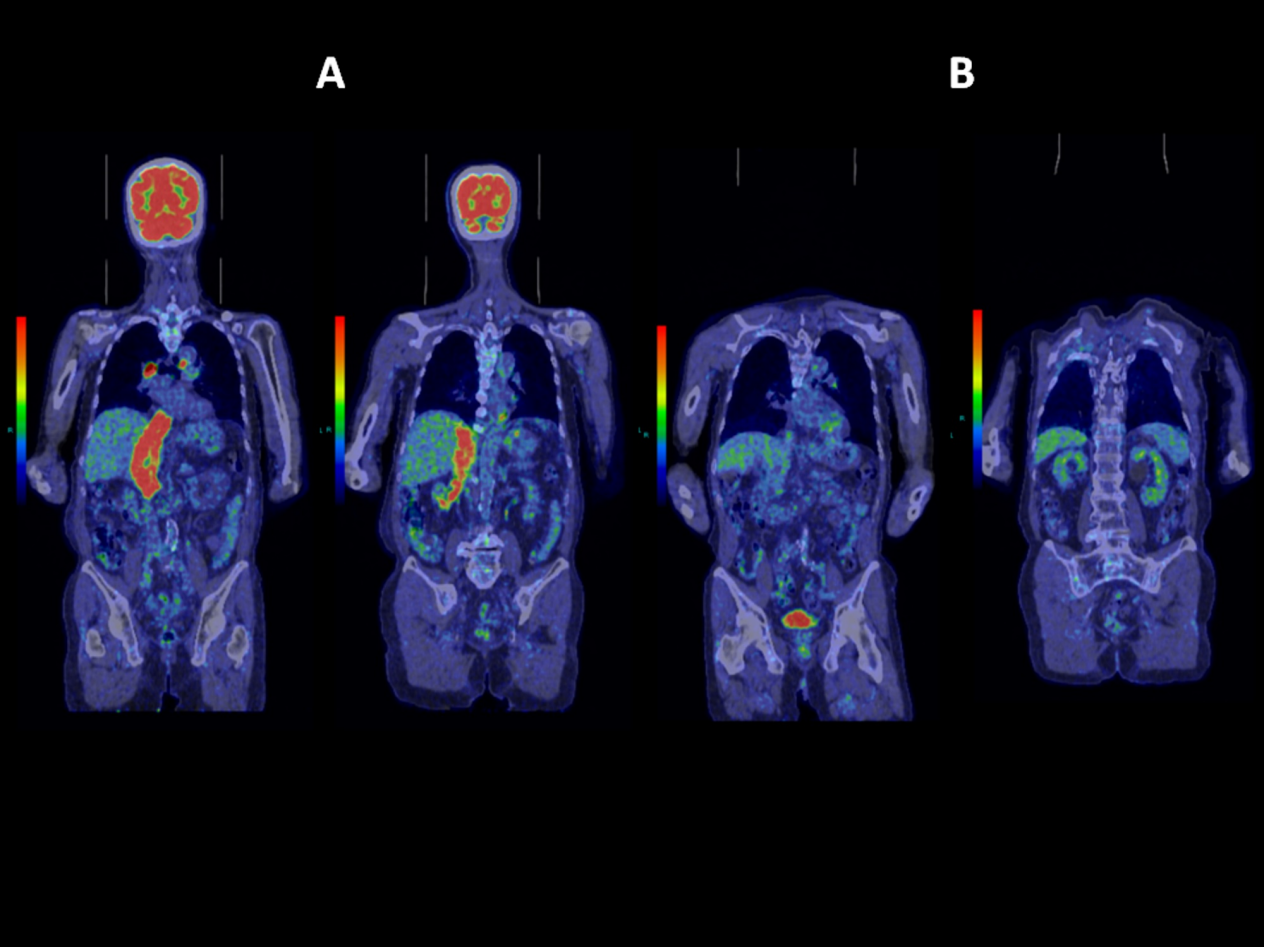
FDG-PET/CT scan before and after treatment. (A) FDG uptake is shown in red spot, revealing IVC tumor, arising from the lower pole of the right kidney, and two lymph node metastases. (B) One year after treatment. FDG uptake is not shown in IVC or kidneys. FDG-PET, fluorodeoxyglucose-positron emission tomography; IVC, inferior vena cava

An 81-year-old male visited our hospital with remarkable edema in both legs, particularly in his right leg. Vascular ultrasound on lower extremities revealed thrombus in the left popliteal vein; however, no thrombus was found in the right leg. Initial laboratory workup revealed mild kidney dysfunction with urea nitrogen level of 28.7 mg/dL, creatinine level of 1.27 mg/dL, and estimated glomerular filtration rate of 42 mL/min. D-dimer was elevated to 8.4 μg/L. Abdominal ultrasound revealed hyper-echoic lesion filled in the IVC from the kidney level to the right atrium. This revealed low-echoic lesion of 23.2 x 23.0 mm in the lower pole of the right kidney, which suggested tumor. Cardiac ultrasound and CT scan of the abdomen with intravenous contrast medium revealed IVC tumor protruding into the right atrium (Figures 1, 2). However, contrast enhancement in kidneys was not clear. FDG-PET (fluorodeoxyglucose-positron emission tomography)/CT scan revealed tumor in the lower pole of the right kidney, which continued into the right renal vein through the IVC (Figure 3). A right mediastinal lymph node and left hilar lymph node also showed FDG uptake. Thus, the patient was diagnosed with IVC tumor embolism possibly of RCC with mediastinal metastasis.

Transperitoneal biopsy was avoided as tumor in the right kidney was small and in the lower pole. Even if biopsy was performed to confirm the diagnosis of RCC, surgical treatment was nearly impossible due to the patient’s age. The patient and his family requested palliative care or minimally invasive treatment to preserve quality of life. CyberKnife was chosen because it is a minimally invasive short-term treatment.

Multisession SRT was performed using CyberKnife G4 system. Tumors were tracked with spine-tracking algorithm. Gross tumor volume (GTV) was defined as visible tumor on enhanced CT scan with images merged for target definition. GTV was considered the same as clinical target volume (CTV). Planning target volume (PTV) included CTV with a 1.2-mm margin.

**Figure 4:**
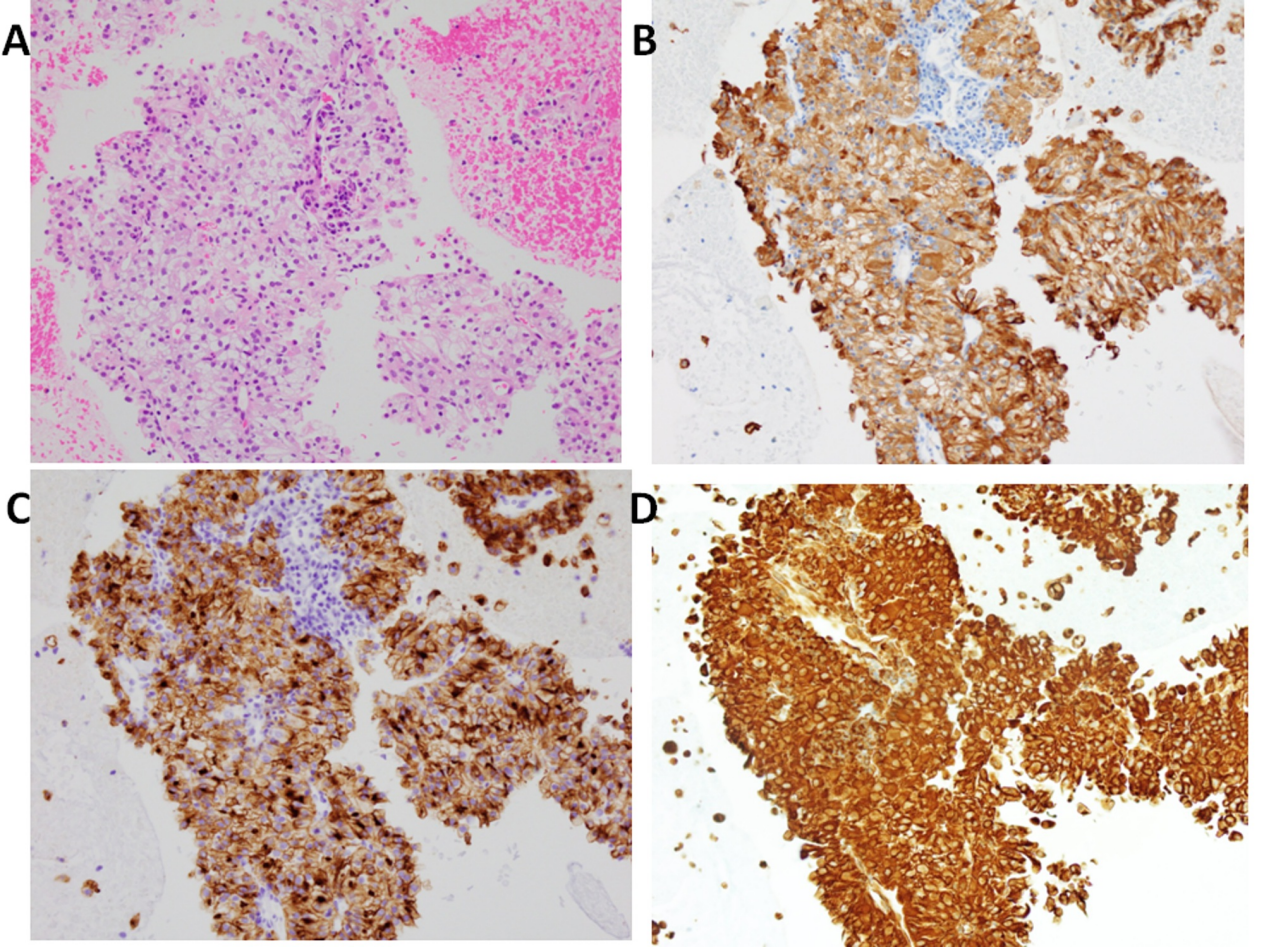
Pathological findings of lymph node biopsy (magnification x20). (A) Hematoxylin-eosin staining. (B) Positive staining with A/E staining. (C) Positive staining with CD 10 staining. (D) Positive staining with  Vimentin. These findings are compatible with clear cell renal cell carcinoma metastatic to lymph node.

Giant IVC tumor (GTV 211.3 cm^3^) was treated with a prescription dose of 40 Gy in 10 fractions and prescription isodose line 89%. Second CyberKnife treatment was performed four months afterward for left hilar lymphadenopathy (GTV: 22.6 cm^3^) with a prescription dose of 40 Gy in five fractions and prescription isodose line 57%. Twelve months after initial treatment, FDG uptake was not observed in IVC or left hilar lymph node. However, right mediastinal lymphadenopathy remained. The patient presented allergic reaction to the contrast medium, thereby precluding its usage. Biopsy was performed by bronchoscope, which revealed clear cell RCC (Figure 4). Eleven months after the second CyberKnife treatment, the third CyberKnife treatment was performed for right mediastinal lymphadenopathy (GTV: 11.7 cm^3^) with a prescription dose of 45 Gy in 10 fractions and prescription isodose line 73%. Cardiac ultrasound did not reveal any mass in the right atrium, which indicated that the tumor in the IVC had shrunk and cancer cells were destroyed over time by radiotherapy.

**Figure 5:**
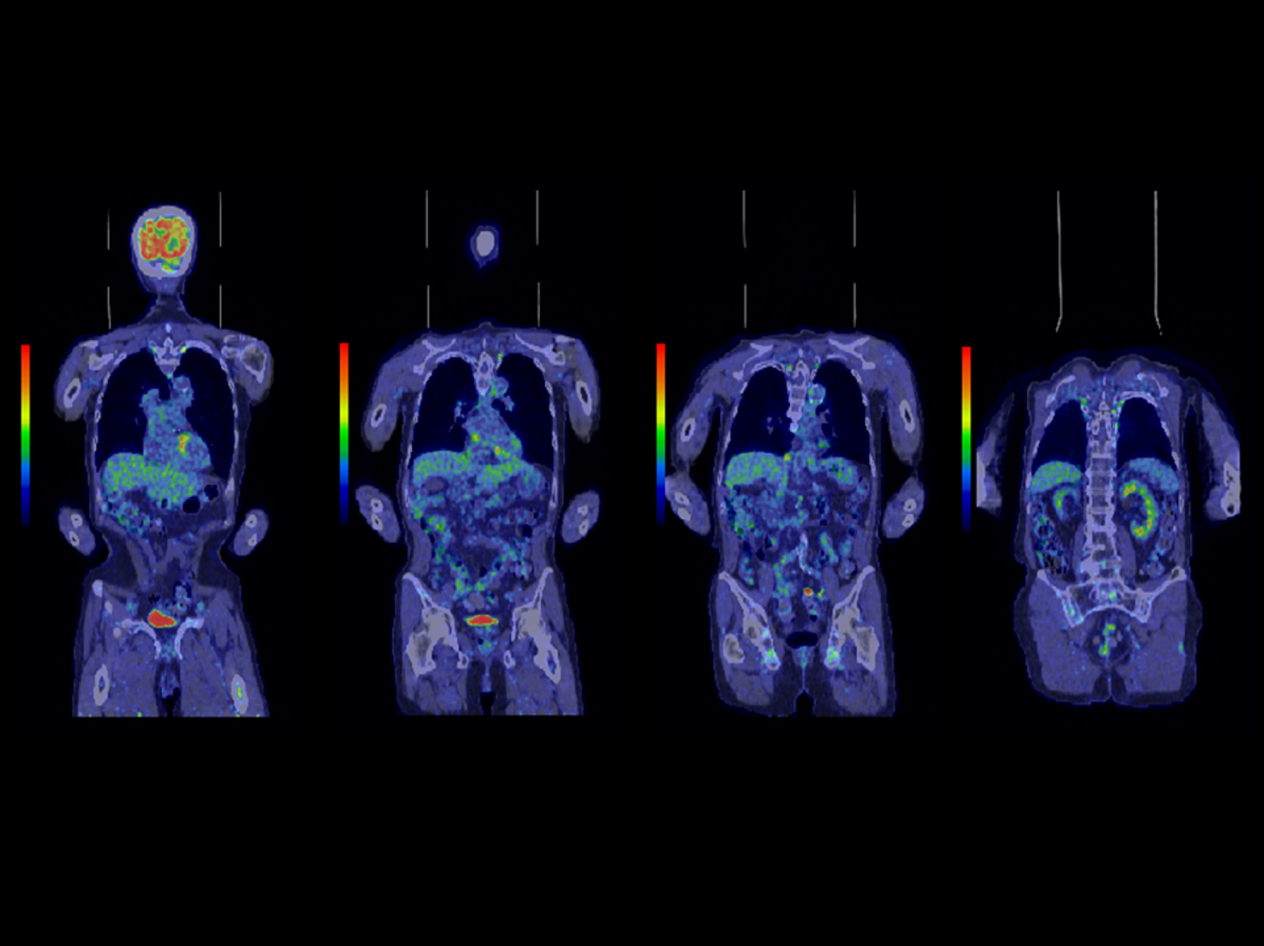
FDG-PET/CT scan three years and three months after initial treatment. Coronal sections from anterior to posterior are displayed from left to right. FDG uptake in IVC tumor and two lymph node metastases have disappeared. FDG-PET, fluorodeoxyglucose-positron emission tomography; IVC, inferior vena cava

All three treatment courses were performed on outpatient basis. Edema in both legs disappeared 18 months after initial treatment. The patient reports no symptoms thereafter. Three years and three months have passed since initial treatment, with FDG-PET indicating cancer remission for IVC tumor and mediastinal lymphadenopathy (Figure 5). The patient is enjoying a normal life without any treatment-related toxicity.

## Discussion

RCC with IVC thrombus above hepatic veins is technically complex, often requiring cardiopulmonary bypass and resulting in a major complication rate of 34% and mortality rate of 10.8% [1]. As tumor thrombi in the IVC protruded into the right atrium, cardiopulmonary bypass was mandatory.

RCC has traditionally been regarded as a radio-resistant tumor based on preclinical data and negative clinical trials using conventional fractionated radiotherapy [2]. However, radiotherapy delivered in few fractions with high single-fraction and total doses may overcome RCCs radio-resistance; thus, SRT has been successful showing high local control and low toxicity [2]. Robotic radiosurgery system is more precise, as Staehler et al. reported that single-fraction radiosurgery using 25 Gy with CyberKnife system resulted in a local control rate of 98% after nine months [3]. In 2018, a multicenter study revealed that single-fraction (median dose of 25 Gy) and multi-fraction (median dose of 40 Gy delivered in 2-10 fractions) stereotactic ablative radiotherapy resulted in a local control rate of 97.8% and a cancer-specific survival rate of 91.9% at four years [4]. Therefore, SRT for RCC is now considered an excellent treatment option for inoperable cases.

In 1987, Didier et al. reported that primary neoplasm causing tumor thrombi of IVC were RCC, adrenal tumors, retroperitoneal tumors, and hepatic tumors [5]. Shuch et al. also reported that vascular invasion commonly occurs in RCC and that the mortality rate of tumor embolism was 75% [6]. The patient survived over one year after urgent life-saving CyberKnife treatment for IVC tumor of unknown origin. Subsequent lymph node biopsy confirmed RCC. Therefore, CyberKnife radiotherapy proved successful in life-saving treatment of massive IVC tumor embolism of RCC.

There have been only two reports of radiotherapy for IVC tumor thrombosis of RCC. One is a case report of intensity-modulated radiotherapy (56 Gy/24 fractions of one fraction per day) that stabilized a tumor embolus in clear cell RCC [7]. Another is the only existing report of SRT for IVC tumor thrombus of RCC by Hannan et al., which showed only partial remission at two years follow-up and 18-month survival [8].

**Figure 6:**
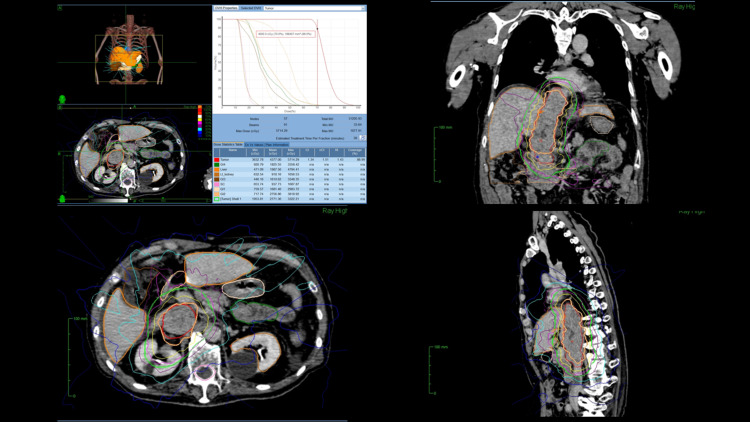
Treatment plan

There is a choice of performing radiotherapy without biopsy or performing chemotherapy according to RCC treatment guidelines because of a higher probability of RCC. CyberKnife radiotherapy was preferable due to its non-invasivity and patient’s poor prognosis. CyberKnife radiotherapy for this patient was appropriately adjusted to treat such a large tumor on an outpatient basis. In standard CyberKnife radiosurgery, a fiducial may be inserted into the tumor to monitor respiratory movement, and fractions are usually three to five. In our case, we avoided insertion of the fiducial into the IVC due to a high risk of bleeding. Instead of inserting the fiducial, treatment plan settings were adjusted to minimize damage by radiation as follows: larger margin for PTV and increased fractions (10 fractions instead of five) (Figure 6). IVC tumor was very closely located to the spine and thus CyberKnife spine-tracking worked efficiently.

## Conclusions

We reported a successful treatment of advanced RCC with massive IVC tumor embolism and lymph node metastases by CyberKnife radiotherapy. The patient is now over 84 years old, surviving without any symptoms, and enjoying normal life. SRT with CyberKnife radiotherapy was successful in treating massive IVC tumor embolism of RCC, which was considered to have a poor prognosis. CyberKnife radiotherapy thereby demonstrates a favorable treatment for elderly patients with IVC tumor thrombus who may otherwise not be good surgical candidates.
